# The Acute Effects Of Hip Thrust and Glute Bridge Exercises With Different Loads on Sprint Performance and Horizontal Force–Velocity Profile in Adolescent Soccer Players: A Post‐Activation Performance Enhancement Approach

**DOI:** 10.1002/ejsc.12255

**Published:** 2025-01-20

**Authors:** Salih Çabuk, İzzet İnce

**Affiliations:** ^1^ Faculty of Sport Sciences Department of Coaching Education Erzurum Technical University Erzurum Türkiye; ^2^ Faculty of Sport Sciences Department of Coaching Education Ankara Yildirim Beyazit University Ankara Türkiye

**Keywords:** glute bridge, hip thrust, horizontal force–velocity profile, post‐activation performance enhancement, sprint

## Abstract

This study examines the acute effects of post‐activation performance enhancement (PAPE) with different loads (84% and 60% 1‐RM) and exercises (hip thrust; HT and glute bridge; GB) on sprint performance (SP) and horizontal force–velocity (HF–V) profile components in adolescent male soccer players. The participants were randomly assigned to three groups: hip thrust group (HTG; *n* = 13), glute bridge group (GBG; *n* = 13), and control group (CG; *n* = 14). Sprint tests at distances of 10, 20, and 30 m were conducted pre‐PAPE and post‐PAPE protocols with a 7 min rest period. HTG and GBG executed HT and GB exercises at 84% and 60% of their 1‐RM loads. Hedge's g was computed to assess within‐group (pre‐PAPE vs. post‐PAPE) comparisons (Within‐ES) and between‐group (post‐PAPE protocols) comparisons (Between‐ES). PAPE protocols at 84% and 60% 1‐RM loads demonstrated moderate effects on F0_rel_, P_maxrel_, FV_slope_, and D_RF_ and small effects on V_0_, RF_max_, and S_20m_ in both HTG and GBG. Conversely, the CG exhibited trivial effects across parameters. Compared to the HTG 84% 1‐RM protocol, the GBG 84% 1‐RM protocol showed small effects on V_0_ and S_10m_. The HTG 60% 1‐RM protocol had a small effect on RF_max_ compared to both GBG 84% and 60% 1‐RM protocols. Both HTG and GBG 84% and 60% 1‐RM protocols demonstrated small effects on S_30m_ compared to the CG. These findings suggest that GB exercises may offer a viable alternative to HT exercises for eliciting PAPE effects, particularly in enhancing SP and related mechanics in adolescent soccer players.

**Summary**

- The hip thrust exercise targets hip extensors, increasing gluteus maximus contribution over hamstrings during hip extension, and is effective for activities needing horizontal force vector such as sprint.
- Researchers often prefer hip thrust exercises for inducing PAPE effects in the horizontal vector. Investigating glute bridge exercises in the same vector could offer new PAPE strategies.
- Our findings suggest that glute bridge exercises might be an alternative to hip thrust exercise for improving sprint performance and kinetics in adolescent soccer players.

## 1 Introduction

Post‐activation potentiation (PAP) refers to an acute performance boost immediately following a preload in explosive movements such as sprint. Factors such as muscle fiber type, fitness level, and conditioning activity (CA) characteristics (type, duration, volume, and intensity) can affect PAP, along with the nature of the subsequent activity (I. Dello Iacono et al. 2018; Petisco et al. 2019). Recently, researchers in the scientific community have debated the distinguishing features of PAP and post‐activation performance enhancement (PAPE). PAP as a short‐term physiological response enhancing muscle twitch through mechanisms such as myosin light chain phosphorylation in type II fibers, whereas PAPE represents a long‐lasting increase in voluntary muscle force production due to factors such as increased muscle temperature and intracellular fluid accumulation. In this context, if a performance‐oriented approach is adopted, it is suggested that the term PAPE is more appropriate than the mechanistic term PAP (Blazevich and Babault 2019; Prieske et al. 2020). Therefore, we have decided that the term PAPE would be suitable for this study. Various athletic parameters, such as vertical jump, bench press throw, and sprint performance (SP), have shown improvements attributed to PAPE effects. To achieve PAPE, a range of CAs has been implemented in different sports, including exercises such as parallel squat, power clean, bench press, plyometrics, eccentric overload movements, sled pull, and hip thrust (HT) (Gautam, Singh, and Varghese 2024; Healy and Comyns 2017; Krzysztofik et al. 2021). Biomechanically, HT exercise induces hip flexion throughout the full range of motion, making it an appropriate exercise for targeting hip extensor muscles (B. Contreras et al. 2011; Kennedy et al. 2022). This exercise also increases the contribution of the gluteus maximus relative to the hamstrings during hip extension. Furthermore, it is highly effective in activities that require force vector along a horizontal vector, such as sprint (Fernández‐Galván et al. 2022). The increasing popularity of the HT exercise among strength and conditioning coaches has led to the development of new variations, such as the glute bridge (GB). The GB exercise involves a hip extension performed with loading along a horizontal vector. The primary difference between these two exercises is the position of the torso during execution (Kennedy et al. 2022). This small difference can alter the entire biomechanical properties of the joints involved in the exercise. The HT exercise elicits greater activation in the vastus lateralis, a key muscle involved in sprint, whereas the GB exercise primarily activates the gluteus medius and gluteus maximus, with the gluteus medius playing a particularly crucial role during the acceleration phase (Howard, Conway, and Harrison 2018; Kennedy et al. 2022; Nuell et al. 2021). Despite the extensive research on the HT as a CA (Carbone et al. 2020; I. Dello Iacono et al. 2018; Dello Iacono and Seitz 2018; Fernández‐Galván et al. 2022), GB remains largely unexplored in this context.

Force, velocity, and power are the primary mechanical characteristics of ballistic movements (P. Samozino et al. 2016). The ability to generate high levels of muscular force and power has been extensively studied as a critical neuromuscular factor for key activities such as acceleration and sprint, which are integral to team sports such as soccer (Dello Iacono and Seitz 2018). The ability to sprint signifies the capacity to generate significant power output in the horizontal plane, which is indicative of pronounced forward acceleration resulting from this power production (J. Morin et al. 2012; Rabita et al. 2015). The capacity to achieve maximum speed and acceleration during sprint is directly associated with the mechanical power linked to the anteroposterior component of ground reaction force. Although SP is influenced by various biomechanical variables, it is commonly assessed based solely on the time taken to cover a specific distance. However, this time‐based approach may limit the full understanding of the mechanical complexities underlying performance and may hinder the identification of muscle's mechanical properties (de Barros Sousa et al. 2024; Pálinkás et al. 2024). Recently, a French research group developed a field method that derives horizontal acceleration data by differentiating the speed‐time curve, enabling the calculation of mechanical outputs, and constructing a horizontal force–velocity (HF–V) profile components for accelerated sprints (Morin and Samozino 2016; P. Samozino et al. 2016). Through this method, key performance components such as relative theoretical maximum force (F_0rel_), theoretical maximum velocity (V_0_), and relative theoretical maximum power (P_maxrel_) can be determined (Rakovic et al. 2018). This approach provides a macroscopic perspective on sprint mechanics, offering a more comprehensive understanding of the dynamics underlying SP (de Barros Sousa et al. 2024; P. Samozino et al. 2018). In the literature, numerous studies have been conducted on HF–V profile components in soccer players (Cross et al. 2018; T. A. Haugen 2018; Jiménez‐Reyes et al. 2018; Marcote‐Pequeño et al. 2019; Mendiguchia et al. 2016). Also, numerous of studies in the literature have investigated the impact of different prior CA on SP in soccer players (Dello Iacono and Seitz 2018; Evetovich, Conley, and McCawley 2015; Mcbride et al. 2005; Petisco et al. 2019; Till and Cooke 2009; Vanderka et al. 2016). It has been suggested that regularly monitoring HF–V profile components in soccer players can provide valuable information for both performance enhancement (Mendiguchia et al. 2016).

Considering these factors, although the HT exercise is commonly preferred to enhance SP by targeting vastus lateralis activation, the GB exercise, which focuses on the gluteus medius and gluteus maximus, may also serve as an effective alternative. These muscles play a crucial role in the acceleration phase of sprint, particularly in sports such as soccer. Both GB and HT exercises engage the horizontal force vector but elicit different muscle activations. This distinction makes it valuable to explore the conditioning potential of the GB exercise at varying loads as compared to the HT. Evaluating the sensitivity of the HF–V profile and the effects of various weighted exercise protocols on its components may suggest that these training regimes could be valuable diagnostic tools for personalized performance analysis. This approach could allow for training plans tailored to an athlete's specific neuromuscular condition, making performance enhancement processes more targeted. Understanding how the relationship between force, velocity, and power shifts can enable a detailed analysis of how muscle neuromuscular properties respond to different load and intensity levels. In this context, evaluating the effects of a CA protocol applied with different loads in two exercises—HT and GB—that share the same horizontal force vector but differ in muscle activation on SP and related kinetic properties would be very meaningful. Such a study could provide important insights for developing more individualized and effective training programs. This study aimed to investigate the acute effects of prior CAs with two different loads (84% and 60% 1‐RM) and exercises (HT and GB) on SP and HF–V profile components in adolescent male soccer players with at least 2 years of resistance training experience.

## 2 Materials and Methods

### 2.1 Participants

The G‐Power analysis conducted to determine the required sample size for the study was based on the following parameters: effect size (Cohen's *f*) = 0.25, 1 − *β* = 0.80, *α* = 0.05, number of groups = 3, and number of measurements = 2. According to the analysis, it was determined that a total of 42 soccer players should be included in the study. Although the study initially commenced with 44 soccer players [hip thrust group (HTG; *n* = 14), glute bridge group (GBG; *n* = 14), and control group (CG; *n* = 16)], four participants withdrew from the study [HTG (*n* = 13), GBG (*n* = 13), and CG (*n* = 14)] due to illness and injuries during the research process. Thus, the study was completed with a total of 40 soccer players (training age: 4.12 ± 1.49 years; strength training age: 2.55 ± 0.75 years; and maturity offset: 1.81 ± 0.73 years). The physical characteristics of these participants are presented in Table 1. The soccer players provided written informed consent after receiving a verbal explanation of the study's purpose, benefits, and potential risks. All procedures were approved by the Institutional Ethics Committee.

**TABLE 1:** Descriptive statistics.

Variables	HTG (*n* = 13)		GBG (*n* = 13)		CG (*n* = 14)		F_(2,37)_	*p*
	X̄	SD	X̄	SD	X̄	SD		
Age (year)	15.61	0.87	15.54	0.78	15.57	0.85	0.028	0.973
Body height (cm)	173.73	7.95	174.21	6.96	173.82	4.70	0.205	0.816
Body weight (kg)	60.06	9.55	61.74	9.38	60.98	5.53	0.133	0.876
Body fat (%)	13.28	4.17	13.43	3.47	13.03	2.30	0.047	0.954
BMI (kg/m^2^)	19.87	2.65	20.08	2.77	20.18	1.62	0.060	0.942
Maturity offset (year)	1.81	0.88	1.84	0.69	1.78	0.66	0.020	0.980

Abbreviations: BMI, body mass index; CG, control group; GBG, glute bridge group; HTG, hip thrust group; *n*, sample size; SD, standard deviation; and X̄, mean.

## 3 Test Procedures

### 3.1 Maturity Offset

The maturational status of the soccer players was estimated using the regression formula presented by Moore et al. (2015) in the literature: $\left[\text{Maturity offset}=-7.99999+(0.0036124\times (\mathrm{age}\times \mathrm{height}))\right]$. 

### 3.2 Sprint Performance and Horizontal Force–Velocity Profile

SP were evaluated with 10, 20 and 30 m sprint tests (S_10m_, S_20m_, and S_30m_, respectively) conducted on a parquet floor. Photocell gates (Microgate, Bolzano, Italy) and standard protocols were used for these tests (Barrera et al. 2023). Each soccer player was given two trial attempts, with a two‐minute active rest period between sprints. Before the sprint tests, air pressure and temperature were measured using a barometer and a temperature monitor (TFA 3006.42). The tests were conducted in a wind‐free environment. The HF–V profile components were calculated according to previously suggested guidelines (P. Samozino et al. 2016). It has been suggested that the S_10m_, S_20m_, and S_30m_ time points are necessary for the performance evaluation of team sport athletes (T. A. Haugen et al. 2020). Before calculating HF–V profile components, the speed‐time curve was modeled using the least squares regression method according to an exponential equation and the best‐fit curve was obtained for each soccer player (P. Samozino et al. 2016).

### 3.3 Load–Velocity Profile and One Repetition Maximum

The 2‐D video analysis to obtain each soccer player's load–velocity profile was conducted according to a previously described procedure (Carzoli et al. 2022). In this analysis, digital images were recorded in slow‐motion mode at 240 frames per second and 1080p HD quality using an iPhone 11. The iPhone 11 was mounted on a tripod 1.50 m away from the area where HT and GB exercises were performed. The obtained footage was analyzed using the Kinovea 0.9.5 software. The individual 1‐RM values for HT and GB exercises for each soccer player were determined according to a previously described procedure (Amara et al. 2021). A linear regression formula (y = a × x + b) was used to determine the 1‐RM based on performances at different loads. In this formula, represents the theoretical minimum average propulsive velocity of 0.20 m/s (González‐Badillo, Marques, and Sánchez‐Medina 2011). Scatter plots were generated for each soccer player to establish the values in the linear equation and a linear regression line was added using Microsoft Excel (version 2304).

#### 3.3.1 PAPE Protocols

In a meta‐analysis (*n* = 47), it was found that individuals with at least 2 years of resistance training experience [effect size (ES) = 0.53] responded more effectively to CAs compared to those with less than 2 years of experience (ES = 0.44). Additionally, it was noted that a recovery period of 5–7 min demonstrated a greater prior CA (ES = 0.62) compared to recovery periods of less than 4 min (ES = 0.15) or more than 8 min (ES = 0.23) (Seitz and Haff 2016). In an another meta‐analysis (*n* = 32), a rest period of 7–8 min is necessary to increase power output in individuals with at least 1 year of resistance training experience. Additionally, this study found that prior CA with moderate loads (60%–84% 1‐RM) showed a greater effect (ES = 1.06) than heavy loads (85%–100% 1‐RM) (ES = 0.31) regardless of training experience (Wilson et al. 2013). The number of repetitions was determined based on a previously study (Fernández‐Galván et al. 2022). The PAPE protocols was designed according to these findings.

### 3.4 Hip Thrust and Glute Bridge Exercises

The soccer players were instructed to follow the HT and GB exercise protocol described in previous studies (B. Contreras et al. 2011; Kennedy et al. 2022) to ensure consistency across all recorded trials, and both HT and GB exercises were performed with the Smith machine. During both exercises, the soccer players were instructed to place their hands on the bar in a comfortable position, with their palms facing their bodies. This grip allowed them to stabilize the bar while keeping their shoulders in an externally rotated position.

### 3.5 Familiarization and Test Sessions

**FIGURE 1:**
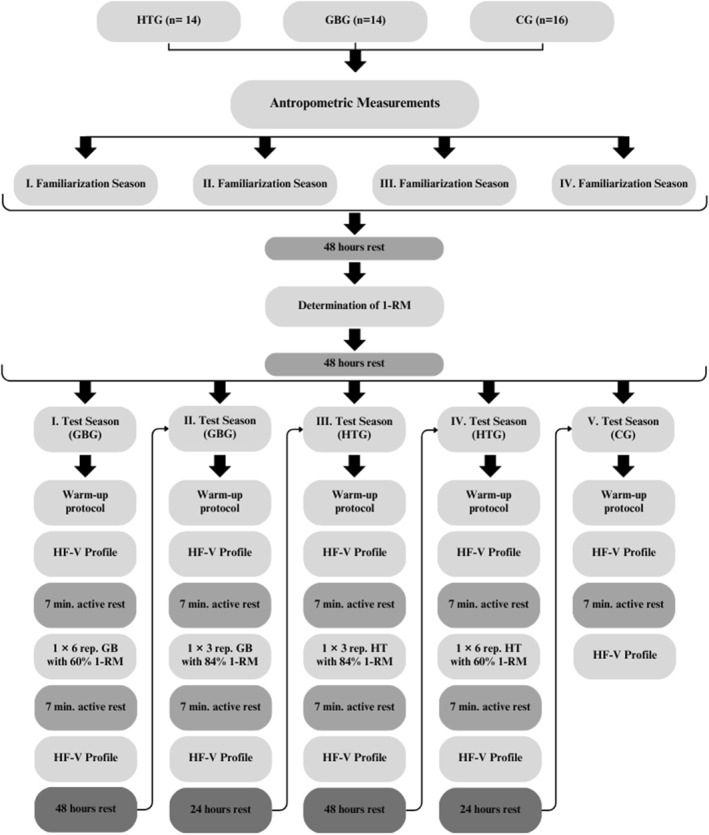
Research design.

All players were randomly assigned to three different groups [HTG (*n* = 14), GBG (*n* = 14), and CG (*n* = 16)]. Following anthropometric measurements, four familiarization sessions were conducted each lasting 45 min. Each session began with a traditional warm‐up and stretching exercises for the lower and upper extremities. In the initial two sessions, soccer players performed HT and GB exercises with a 20 kg (bar's weight), completing 2 sets of 10 and 2 sets of 15 repetitions, with 1 min rest between sets and 3 min rest between different loads. In the third session, they performed 2 sets of 10 repetitions with free weights, 2 sets of 10 repetitions with 25 kg, and 2 sets of 8 repetitions with 30 kg. The final session included 3 sets of 8 repetitions with 25 kg and 2 sets of 8 repetitions with 30 kg, with 2 min rest between sets and 5 min rest between different loads. Subsequently, individual 1‐RM values were determined, with 48 h rest periods before and after to minimize fatigue. Players were instructed to maintain regular diet and sleep routines and avoid additional training. The 1‐RM values were determined using the load–velocity profile protocol, after which test sessions began. During test sessions, players performed preload exercises at 84% and 60% of their 1‐RM. Following a standard warm‐up, they performed dynamic and static stretches and then completed one set of eight repetitions of HT and GB exercises with 20 and 30 kg loads. In the first test session, players in the GBG performed two repetitions of the 10, 20, and 30 m sprints with 2 min rest. They rested for 7 min, performed the GB exercise with six repetitions at 60% 1‐RM, rested again for 7 min, and then repeated the sprint tests. The second session followed the same protocol, but with GB at 84% 1‐RM for three repetitions. In the third and fourth sessions, players in HTG performed the HT exercise at 84% and 60% 1‐RM for three and six repetitions, respectively. In the fifth session, the CG group performed the sprint tests, rested for 7 min, and repeated the sprints (Figure 1). This study was designed to include both within‐subject and between‐subject comparisons, allowing for the effects of PAPE protocols to be evaluated by considering individual variability and differences between groups. All players maintained their regular training routines and engaged in active standing rest during 7 min rest periods. Measurements were conducted between 16:00 and 18:00 to avoid circadian rhythm effects. The same 20 kg barbell and bumper plates were used throughout both familiarization and test sessions to ensure consistency.

### 3.6 Statistical Analysis

Data are presented as mean ± standard deviation (X̄ ± SD) and 95% confidence intervals (95% CI). A one‐way ANOVA was used to examine pre‐intervention differences among groups. A three‐way mixed‐design repeated measures ANOVA (Group = 3 × Protocol = 2 × Time = 2) applied the effects of the intervention and differences between groups. Effect size was reported as partial eta squared (*η*_p_^2^) with thresholds: small > 0.01, medium ≥ 0.06, and large ≥ 0.14. Sphericity assumption was assessed using Mauchly's test. If the sphericity assumption was violated and Epsilon (*ε*) < 0.75, the Greenhouse–Geisser correction was applied. If *ε* > 0.75, the Huynh–Feldt correction was used. Changes in within‐group (pre‐PAPE vs. post‐PAPE) comparisons (Within‐ES) and between‐group (post‐PAPE protocols) comparisons (Between‐ES) were summarized using Hedge's g and classified as trivial (0.00–0.19), small (0.20–0.59), moderate (0.60–1.19), large (1.20–1.99), and very large (≥ 2.00). Intraclass correlation coefficient (ICC) was calculated and summarized with 95% confidence intervals. The ICC's were based on the mean rating, consistency, and two‐way mixed‐effects model. Although ICC's values can be high when there are large differences between participants despite low consistency between trials, the standard error of measurement (SEM) is not affected by the variability between participants (Hermassi, Laudner, and Schwesig 2020). In this context, SEM was calculated using the formula: $\text{standar}d\;\text{deviation}\;x\;\sqrt{\left(1-\text{ICC}\right)}$. The coefficient of variation (CV) was determined using the formula: SEM/mean×100. Percentage changes (%Δ) between times for the groups were also computed. The significance level was set at *α* < 0.05.

## 4 Results

1‐RM for the HTG was 105.58 ± 17.03 kg (range: 69.86–122.97 kg), and for the GBG, it was 113.07 ± 19.21 kg (range: 85.70–136.72 kg).

No statistically significant differences were found between the groups in terms of age, body height, body weight, body fat percentage, BMI, and maturity offset (*p* > 0.05). Additionally, no statistically significant differences were found between the groups in both protocols for F_0rel_, V_0_, P_maxrel_, FV_slope_, RF_max_, D_RF_, S_10m_, S_20m_, and S_30m_ before PAPE protocols (*p* > 0.05) (Table 1).

The time main effect (*F*_(1,37)_ = 35.735 and *η*_p_^2^ = 0.491; *F*_(1,37)_ = 35.385 and *η*_p_^2^ = 0.489; *F*_(1,37)_ = 36.316 and *η*_p_^2^ = 0.495; *F*_(1,37)_ = 36.186 and *η*_p_^2^ = 0.494; *F*_(1,37)_ = 34.279 and *η*_p_^2^ = 0.481; and all *p* < 0.001 and *F*_(1,37)_ = 6.943, *p* = 0.012, and *η*_p_^2^ = 0.158, respectively) and group × time interaction effects (*F*_(2,37)_ = 11.041 and *η*_p_^2^ = 0.374; *F*_(2,37)_ = 12.781 and *η*_p_^2^ = 0.409; *F*_(2,37)_ = 9.868 and *η*_p_^2^ = 0.348; *F*_(2,37)_ = 9.612 and *η*_p_^2^ = 0.342; *F*_(2,37)_ = 22.755 and *η*_p_^2^ = 0.552; *F*_(2,37)_ = 16.085 and *η*_p_^2^ = 0.465; and all *p* < 0.001, respectively) were statistically significant for F_0rel_, P_maxrel_, FV_slope_, D_RF_, S_10m_, and S_30m_. Additionally, the time main effect was significant for V_0_, RF_max_, and S_20m_ (*F*_(1,37)_ = 19.643, *p* < 0.001, and *η*_p_^2^ = 0.347; *F*_(1,37)_ = 4.346, *p* = 0.044, and *η*_p_^2^ = 0.105; F_(1,37)_ = 9.955, *p* = 0.003, and *η*_p_^2^ = 0.212, respectively). There were no statistically significant effects for the group and protocol main effect nor for the group × protocol, time × protocol, and group × time × protocol interaction effects (*p* > 0.05).

Based on the ICC, SEM, and CV results (Table 2), in the 84% 1‐RM protocol, the ICC for HTG and GBG ranges from 0.705 (0.033–0.901) to 0.991 (0.972–0.997). SEM values range from 0.013 to 0.936 and CV values range from 0.318 to 8.188. For the 60% 1‐RM protocol, ICC ranges from 0.844 (0.487–0.952) to 0.986 (0.955–0.996), SEM ranges from 0.015 to 0.902, and CV ranges from 0.367 to 5.949.

**TABLE 2:** ICC, SEM, and CV coefficients for different protocols within groups over time.

Variables	%84 1‐RM						%60 1‐RM						CG		
	HTG			GBG			HTG			GBG					
	ICC (%95 CI)	SEM	CV	ICC (%95 CI)	SEM	CV	ICC (%95 CI)	SEM	CV	ICC (%95 CI)	SEM	CV	ICC (%95 CI)	SEM	CV
F_0rel_ (N/kg)	0.705 (0.033–0.910)	0.936	8.188	0.943 (0.814–0.983)	0.435	3.761	0.863 (0.550–0.958)	0.519	4.446	0.900 (0.674–0.970)	0.580	5.102	0.930 (0.783–0.978)	0.462	4.293
V_0_ (m/s)	0.975 (0.919–0.992)	0.051	0.697	0.983 (0.943–0.995)	0.047	0.641	0.913 (0.713–0.973)	0.118	1.608	0.929 (0.768–0.978)	0.110	1.485	0.978 (0.933–0.993)	0.054	0.726
P_maxrel_ (W/kg)	0.902 (0.678–0.970)	0.844	3.965	0.943 (0.814–0.983)	0.720	3.386	0.904 (0.685–0.971)	0.732	3.427	0.908 (0.697–0.972)	0.902	4.301	0.944 (0.825–0.982)	0.736	3.705
FV_slope_ (N.s.m^−1^.kg^−1^)	0.860 (0.542–0.957)	0.091	5.708	0.947 (0.825–0.984)	0.066	4.185	0.846 (0.496–0.953)	0.090	5.657	0.905 (0.689–0.971)	0.091	5.892	0.930 (0.783–0.978)	0.072	4.937
RF_max_ (%)	0.899 (0.670–0.969)	0.516	1.121	0.890 (0.639–0.966)	0.495	1.079	0.931 (0.774–0.979)	0.393	0.852	0.896 (0.660–0.968)	0.513	1.116	0.954 (0.856–0.985)	0.396	0.867
D_RF_ (%)	0.854 (0.522–0.955)	0.876	5.865	0.946 (0.822–0.938)	0.626	4.234	0.844 (0.487–0.952)	0.859	5.738	0.904 (0.685–0.971)	0.861	5.949	0.932 (0.788–0.978)	0.675	4.919
S_10m_ (s)	0.976 (0.921–0.993)	0.013	0.666	0.952 (0.842–0.985)	0.018	0.900	0.908 (0.698–0.972)	0.020	1.014	0.926 (0.758–0.977)	0.024	1.193	0.961 (0.878–0.987)	0.017	0.837
S_20m_ (s)	0.987 (0.958–0.996)	0.014	0.413	0.979 (0.930–0.994)	0.017	0.501	0.986 (0.955–0.996)	0.015	0.444	0.973 (0.913–0.992)	0.021	0.598	0.903 (0.698–0.969)	0.048	1.392
S_30m_ (s)	0.986 (0.954–0.996)	0.019	0.399	0.991 (0.972–0.997)	0.015	0.318	0.986 (0.953–0.996)	0.018	0.367	0.977 (0.924–0.993)	0.026	0.538	0.988 (0.961–0.996)	0.021	0.430

Abbreviations: %60 1‐RM, 60% of 1 repetition maximum; %84 1‐RM, 84% of 1 repetition maximum; 95% CI, 95% confidence interval; CG, control group; CV, coefficient of variation; D_RF_, decrease in horizontal force; F_0rel_, relative theoretical maximum force; FV_slope_, slope of the force–velocity relationship; GBG, glute bridge group; HTG, hip thrust group; ICC, intraclass correlation coefficient; P_maxrel_, relative theoretical maximum power; RF_max_, maximal horizontal force ratio; S_10m_, 10‐meter sprint performance; S_20m_, 20‐meter sprint performance; S_30m_, 30‐meter sprint performance; SEM, standard error of measurements; and V_0,_ theoretical maximum velocity.

**FIGURE 2:**
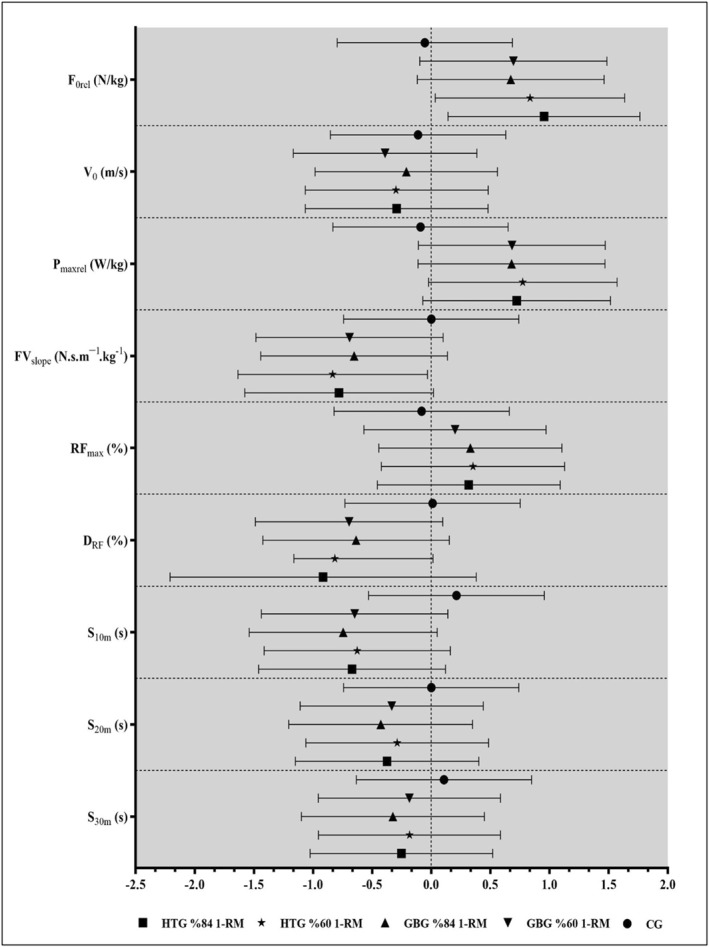
Effect sizes (Hedge's g) for within‐group (pre‐PAPE vs. post‐PAPE) comparisons (Within‐ES).

According to the results of within‐ES comparisons (Table 3), HTG and GBG 84% and 60% 1‐RM protocols had a moderate effect on F_0rel_ (%Δ = 13.54 to 19.50 and *g* = 0.695 to 0.954), P_maxrel_ (%Δ = 12.05 to 13.11 and *g* = 0.679 to 0.774), FV_slope_ (%Δ = 15.54 to 16.90 and *g* = −0.652 to −0.834), D_RF_ (%Δ = 15.24 to 17.10 and *g* = − 0.636 to ‐ 0.915), and S_10m_ (%Δ = − 2.48 to ‐ 2.97 and *g* = −0.626 to −0.745) and a small effect on V_0_ (%Δ = −1.08 to −2.00 and *g* = −0.211 to −0.390), RF_max_ (%Δ = 0.67 to 1.18 and *g* = 0.201 to 0.353), and S_20m_ (%Δ = −1.16 to −1.45 and *g* = −0.288 to −0.427). Conversely, in the CG, F_0rel_, V_0_, P_maxrel_, FV_slope_, RF_max_, D_RF_, S_20m_, and S_30m_ values obtained 7 min before and after showed trivial effects (Figure 2).

**TABLE 3:** Within‐ES and between‐ES comparisons.

Variables	Group	Protocols	Pre‐PAPE		Post‐PAPE		%Δ	Within‐ES	Between‐ES	
			X̄ (95% CI)	SD	X̄ (95% CI)	SD		Hedge's g (95% CI)	Protocols	Hedge's g (95% CI)
F_0rel_ (N/kg)	HTG	%84 1‐RM	10.41 (9.37 – 11.45)	1.723	12.44 (10.95 – 13.93)	2.468	19.50	0.954 (0.142 – 1.765)	HTG %84 1‐RM versus HTG %60 1‐RM	−0.009 (‐0.778 – 0.760)
									HTG %84 1‐RM versus GBG %84 1‐RM	−0.048 (−0.816 – 0.721)
		%60 1−RM	10.93 (10.08 – 11.78)	1.402	12.42 (11.15 – 13.69)	2.096	13.63	0.836 (0.034 – 1.637)	HTG %84 1‐RM versus GBG %60 1‐RM	−0.124 (−0.893 – 0.646)
									HTG %84 1‐RM versus CG	−0.787 (−1.570 – ‐ 0.003)
	GBG	%84 1‐RM	10.82 (9.72 – 11.93)	1.823	12.32 (10.76 – 13.87)	2.578	13.86	0.672 (−0.118 – 1.462)	HTG %60 1‐RM versus GBG %84 1‐RM	−0.043 (−0.811 – 0.726)
									HTG %60 1‐RM versus GBG %60 1‐RM	−0.124 (−0.894 – 0.645)
		%60 1‐RM	10.59 (9.48 – 11.70)	1.833	12.13 (10.59 – 13.66)	2.543	13.54	0.695 (‐0.097 – 1.486)	HTG %60 1‐RM versus CG	−0.854 (−1.643 – ‐ 0.065)
									GBG %84 1‐RM versus GBG %60 1‐RM	−0.074 (−0.843 – 0.695)
	CG	7 min rest	10.82 (9.81 – 11.83)	1.748	10.72 (9.63 – 11.81)	1.888	−0.92	−0.055 (−0.796 – 0.686)	GBG %84 1‐RM versus CG	−0.712 (−1.491 – 0.066)
									GBG %60 1‐RM versus CG	−0.633 (−1.407 – 0.140)
V_0_ (m/s)	HTG	%84 1‐RM	7.37 (7.17 – 7.56)	0.323	7.28 (7.10 – 7.45)	0.291	−1.22	−0.293 (−1.066 – 0.480)	HTG %84 1‐RM versus HTG %60 1‐RM	0.031 (−0.738 – 0.800)
									HTG %84 1‐RM versus GBG %84 1‐RM	0.201 (−0.569 – 0.972)
		%60 1‐RM	7.40 (7.15 – 7.64)	0.400	7.29 (7.07 – 7.50)	0.351	−1.49	−0.299 (−1.065 – 0.481)	HTG %84 1‐RM versus GBG %60 1‐RM	0.185 (−0.585 – 0.956)
									HTG %84 1‐RM versus CG	0.276 (‐0.482 – 1.035)
	GBG	%84 1‐RM	7.43 (7.21 – 7.65)	0.363	7.35 (7.11 – 7.59)	0.396	−1.08	−0.211 (‐0.982 – 0.560)	HTG %60 1‐RM versus GBG %84 1‐RM	0.160 (−0.610 – 0.930)
									HTG %60 1‐RM versus GBG %60 1‐RM	0.142 (−0.628 – 0.912)
		%60 1‐RM	7.49 (7.24 – 7.74)	0.413	7.34 (7.13 – 7.55)	0.353	−2.00	−0.390 (−1.167 – 0.386)	HTG %60 1‐RM versus CG	0.227 (−0.531 – 0.984)
									GBG %84 1‐RM versus GBG %60 1‐RM	−0.027 (−0.795 – 0.742)
	CG	7 min rest	7.41 (7.20 – 7.62)	0.362	7.37 (7.17 – 7.58)	0.355	−0.54	−0.112 (−0.853 – 0.630)	GBG %84 1‐RM versus CG	0.053 (−0.702 – 0.808)
									GBG %60 1‐RM versus CG	0.085 (−0.671 – 0.840)
P_maxrel_ (W/kg)	HTG	%84 1‐RM	19.98 (18.35 – 21.61)	2.697	22.60 (19.96 – 25.23)	4.361	13.11	0.723 (−0.071 – 1.516)	HTG %84 1‐RM versus HTG %60 1‐RM	−0.002 (−0.771 – 0.766)
									HTG %84 1‐RM versus GBG %84 1‐RM	−0.028 (−0.797 – 0.741)
		%60 1‐RM	20.16 (18.73 – 21.59)	2.360	22.59 (20.31 – 24.86)	3.760	12.05	0.774 (−0.023 – 1.571)	HTG %84 1‐RM versus GBG %60 1‐RM	−0.099 (−0.869 – 0.670)
									HTG %84 1‐RM versus CG	−0.756 (−1.537 – 0.025)
	GBG	%84 1‐RM	20.03 (18.21 – 21.86)	3.015	22.48 (19.99 – 24.97)	4.116	12.23	0.679 (−0.112 – 1.470)	HTG %60 1‐RM versus GBG %84 1‐RM	−0.028 (−0.797 – 0.741)
									HTG %60 1‐RM versus GBG %60 1‐RM	−0.104 (−0.874 – 0.665)
		%60 1‐RM	19.74 (17.94 – 21.53)	2.972	22.18 (19.71 – 24.65)	4.089	12.36	0.683 (−0.108 – 1.471)	HTG %60 1‐RM versus CG	−0.822 (−1.608 – ‐ 0.036)
									GBG %84 1‐RM versus GBG %60 1‐RM	−0.073 (−0.842 – 0.696)
	CG	7 min rest	20.00 (18.21 – 21.80)	3.109	19.71 (17.84 – 21.59)	3.247	−1.45	−0.091 (−0.832 – 0.650)	GBG %84 1‐RM versus CG	−0.751 (−1.532 – 0.030)
									GBG %60 1‐RM versus CG	−0.672 (−1.448 – 0.104)
FV_slope_ (N.s.m^−1^.kg^−1^)	HTG	%84 1‐RM	−1.47 (−1.62 – ‐1.33)	0.243	−1.71 (−1.93 – ‐1.50)	0.361	16.33	−0.780 (−1.577 – 0.018)	HTG %84 1‐RM versus HTG %60 1‐RM	0.001 (−0.769 – 0.769)
									HTG %84 1‐RM versus GBG %84 1‐RM	0.052 (−0.717 – 0.821)
		%60 1‐RM	−1.48 (−1.62 – ‐1.34)	0.230	−1.71 (−1.90 – ‐1.52)	0.315	15.54	−0.834 (−1.635 – ‐ 0.032)	HTG %84 1‐RM versus GBG %60 1‐RM	0.133 (−0.637 – 0.902)
									HTG %84 1‐RM versus CG	0.759 (−0.022 – 1.541)
	GBG	%84 1‐RM	−1.46 (−1.64 – ‐1.29)	0.287	−1.69 (−1.94 – ‐1.44)	0.408	15.75	−0.652 (−1.441 – 0.137)	HTG %60 1‐RM versus GBG %84 1‐RM	0.055 (−0.714 – 0.824)
									HTG %60 1‐RM versus GBG %60 1‐RM	0.140 (−0.629 – 0.910)
		%60 1‐RM	−1.42 (−1.60 – ‐1.24)	0.295	−1.66 (−1.90 – ‐1.43)	0.393	16.90	−0.691 (−1.482 – 0.101)	HTG %60 1‐RM versus CG	0.818 (0.032 – 1.603)
									GBG %84 1‐RM versus GBG %60 1‐RM	0.075 (−0.694 – 0.844)
	CG	7 min rest	−1.46 (−1.62 – ‐ 1.31)	0.273	−1.46 (−1.63 – ‐ 1.29)	0.297	0.00	0.001 (−0.741 – 0.741)	GBG %84 1‐RM versus CG	0.649 (−0.126 – 1.423)
									GBG %60 1‐RM versus CG	0.577 (−0.193 – 1.348)
RF_max_ (%)	HTG	%84 1‐RM	45.85 (44.86 – 46.83)	1.625	46.31 (45.44 – 47.06)	1.251	1.00	0.317 (−0.456 – 1.091)	HTG %84 1‐RM versus HTG %60 1‐RM	0.106 (−0.663 – 0.875)
									HTG %84 1‐RM versus GBG %84 1‐RM	−0.126 (−0.896 – 0.643)
		%60 1‐RM	45.92 (45.02 – 46.83)	1.498	46.46 (45.52 – 47.40)	1.561	1.18	0.353 (−0.422 – 1.128)	HTG %84 1‐RM versus GBG %60 1‐RM	−0.167 (−0.937 – 0.603)
									HTG %84 1‐RM versus CG	−0.426 (−1.189 – 0.338)
	GBG	%84 1‐RM	45.69 (44.79 – 46.59)	1.493	46.15 (45.38 – 46.93)	1.281	1.01	0.331 (−0.443 – 1.105)	HTG %60 1‐RM versus GBG %84 1‐RM	−0.217 (−0.988 – 0.554)
									HTG %60 1‐RM versus GBG %60 1‐RM	−0.248 (−1.020 – 0.523)
		%60 1‐RM	45.77 (44.81 – 46.73)	1.589	46.08 (45.17 – 46.98)	1.498	0.67	0.201 (−0.570 – 0.971)	HTG %60 1‐RM versus CG	−0.482 (−1.247 – 0.284)
									GBG %84 1‐RM versus GBG %60 1‐RM	−0.050 (−0.819 – 0.719)
	CG	7 min rest	45.79 (44.72 – 46.82)	1.847	45.64 (44.59 – 46.70)	1.823	−0.33	−0.082 (−0.823 – 0.659)	GBG %84 1‐RM versus CG	−0.322 (−1.081 – 0.438)
									GBG %60 1‐RM versus CG	−0.263 (−1.021 – 0.495)
D_RF_ (%)	HTG	%84 1‐RM	−13.80 (−15.19–‐12.42)	2.293	−16.07 (−18.13–‐14.02)	3.401	16.45	−0.915 (−2.209–0.380)	HTG %84 1‐RM versus HTG %60 1‐RM	0.013 (−0.756–0.781)
									HTG %84 1‐RM versus GBG %84 1‐RM	0.063 (−0.706–0.832)
		%60 1‐RM	−13.91 (−15.22–‐12.59)	2.174	−16.03 (−17.82–‐14.23)	2.969	15.24	−0.815 (−1.615–0.015)	HTG %84 1‐RM versus GBG %60 1‐RM	0.129 (−0.640–0.899)
									HTG %84 1‐RM versus CG	0.764 (−0.018–1.545)
	GBG	%84 1‐RM	−13.72 (−15.35–‐12.09)	2.693	−15.84 (−18.18–‐13.50)	3.871	15.45	−0.636 (−1.424–0.152)	HTG %60 1‐RM versus GBG %84 1‐RM	0.055 (−0.714–0.824)
									HTG %60 1‐RM versus GBG %60 1−RM	0.125 (−0.645–0.894)
		%60 1‐RM	−13.33 (−15.01–‐11.65)	2.779	−15.61 (−17.85–‐13.36)	3.715	17.10	−0.695 (−1.487–0.097)	HTG %60 1‐RM versus CG	0.808 (0.023–1.593)
									GBG %84 1‐RM versus GBG %60 1‐RM	0.061 (−0.708–0.830)
	CG	7 min rest	−13.73 (−15.22–‐12.24)	2.587	−13.70 (−15.32–‐12.08)	2.802	−0.22	0.011 (−0.730–0.752)	GBG %84 1‐RM versus CG	0.637 (−0.137–1.411)
									GBG %60 1‐RM versus CG	0.584 (−0.187–1.355)
S_10m_ (s)	HTG	%84 1‐RM	2.04 (1.98–2.09)	0.086	1.98 (1.92–2.03)	0.093	−2.94	−0.670 (−1.460–0.120)	HTG %84 1‐RM versus HTG %60 1‐RM	−0.109 (−0.878–0.661)
									HTG %84 1‐RM versus GBG %84 1‐RM	−0.232 (−1.003–0.540)
		%60 1‐RM	2.02 (1.98–2.06)	0.067	1.97 (1.91–2.02)	0.091	−2.48	−0.626 (−1.413–0.162)	HTG %84 1‐RM versus GBG %60 1‐RM	−0.105 (−0.875–0.664)
									HTG %84 1‐RM versus CG	0.720 (−0.059–1.499)
	GBG	%84 1‐RM	2.02 (1.97–2.07)	0.082	1.96 (1.92–2.01)	0.079	−2.97	−0.745 (−1.540–0.050)	HTG %60 1‐RM versus GBG %84 1‐RM	−0.117 (−0.887–0.652)
									HTG %60 1‐RM versus GBG %60 1‐RM	0.001 (−0.769–0.769)
		%60 1‐RM	2.03 (1.98–2.08)	0.088	1.97 (1.91–2.03)	0.097	−2.96	−0.648 (−1.437–0.141)	HTG %60 1‐RM versus CG	0.832 (0.031–1.634)
									GBG %84 1‐RM versus GBG %60 1‐RM	0.113 (−0.656–0.882)
	CG	7 min rest	2.03 (1.98–2.08)	0.086	2.05 (1.99–2.10)	0.101	0.98	0.213 (−0.530–0.956)	GBG %84 1‐RM versus CG	0.988 (0.188–1.787)
									GBG %60 1‐RM versus CG	0.807 (0.022–1.592)
S_20m_ (s)	HTG	%84 1‐RM	3.44 (3.37–3.52)	0.124	3.39 (3.30–3.48)	0.143	−1.45	−0.374 (−1.149–0.402)	HTG %84 1‐RM versus HTG %60 1‐RM	0.001 (−0.769–0.769)
									HTG %84 1‐RM versus GBG %84 1‐RM	0.077 (−0.692–0.846)
		%60 1‐RM	3.43 (3.36–3.51)	0.128	3.39 (3.30–3.48)	0.149	−1.17	−0.288 (−1.061–0.485)	HTG %84 1‐RM versus GBG %60 1‐RM	0.155 (−0.615–0.925)
									HTG %84 1‐RM versus CG	0.412 (−0.351–1.175)
	GBG	%84 1‐RM	3.45 (3.38–3.52)	0.118	3.40 (3.33–3.47)	0.116	−1.45	−0.427 (−1.205–0.350)	HTG %60 1‐RM versus GBG %84 1‐RM	0.075 (−0.694–0.844)
									HTG %60 1‐RM versus GBG %60 1‐RM	0.151 (−0.619–0.921)
		%60 1‐RM	3.45 (3.48–3.53)	0.125	3.41 (3.34–3.48)	0.114	−1.16	−0.334 (−1.109–0.440)	HTG %60 1‐RM versus CG	0.404 (−0.358–1.167)
									GBG %84 1‐RM versus GBG %60 1‐RM	0.087 (−0.682–0.856)
	CG	7 min rest	3.45 (3.36–3.54)	0.154	3.45 (3.37–3.54)	0.148	0.00	0.001 (−0.741–0.741)	GBG %84 1‐RM versus CG	0.374 (−0.387–1.136)
									GBG %60 1‐RM versus CG	0.301 (−0.458–1.060)
S_30m_ (s)	HTG	%84 1‐RM	4.85 (4.75–4.95)	0.163	4.81 (4.72–4.90)	0.154	−0.83	−0.252 (‐1.024–0.520)	HTG %84 1‐RM versus HTG %60 1‐RM	−0.060 (−0.829–0.708)
									HTG %84 1‐RM versus GBG %84 1‐RM	−0.199 (−0.970–0.571)
		%60 1‐RM	4.83 (4.74–4.92)	0.149	4.80 (4.69–4.91)	0.176	−0.62	−0.184 (−0.954–0.586)	HTG %84 1‐RM versus GBG %60 1‐RM	−0.130 (−0.899–0.640)
									HTG %84 1‐RM versus CG	0.295 (−0.464–1.054)
	GBG	%84 1‐RM	4.83 (4.73–4.92)	0.161	4.78 (4.70–4.87)	0.147	−1.04	−0.324 (−1.098–0.449)	HTG %60 1‐RM versus GBG %84 1‐RM	−0.123 (−0.893–0.646)
									HTG %60 1‐RM versus GBG %60 1‐RM	−0.060 (−0.829–0.708)
		%60 1‐RM	4.82 (4.72–4.93)	0.170	4.79 (4.70–4.89)	0.154	−0.62	−0.185 (−0.955–0.585)	HTG %60 1‐RM versus CG	0.334 (−0.426–1.094)
									GBG %84 1‐RM versus GBG %60 1‐RM	0.066 (−0.703–0.835)
	CG	7 min rest	4.84 (4.73–4.95)	0.190	4.86 (4.76–4.97)	0.183	0.41	0.107 (−0.634–0.849)	GBG %84 1‐RM versus CG	0.480 (−0.286–1.246)
									GBG %60 1‐RM versus CG	0.412 (−0.350–1.175)

Abbreviations: %60 1‐RM, 60% of 1 repetition maximum; %84 1‐RM, 84% of 1 repetition maximum; 95% CI, 95% confidence interval; CG, control group; D_RF_, decrease in horizontal force; F_0rel_, relative theoretical maximum force; FV_slope_, slope of the force–velocity relationship; GBG, glute bridge group; HTG, hip thrust group; P_maxrel_, relative theoretical maximum power; RF_max_, maximal horizontal force ratio; S_10m_, 10‐meter sprint performance; S_20m_, 20‐meter sprint performance; S_30m_, 30‐meter sprint performance; and V_0,_ theoretical maximum velocity.

**FIGURE 3:**
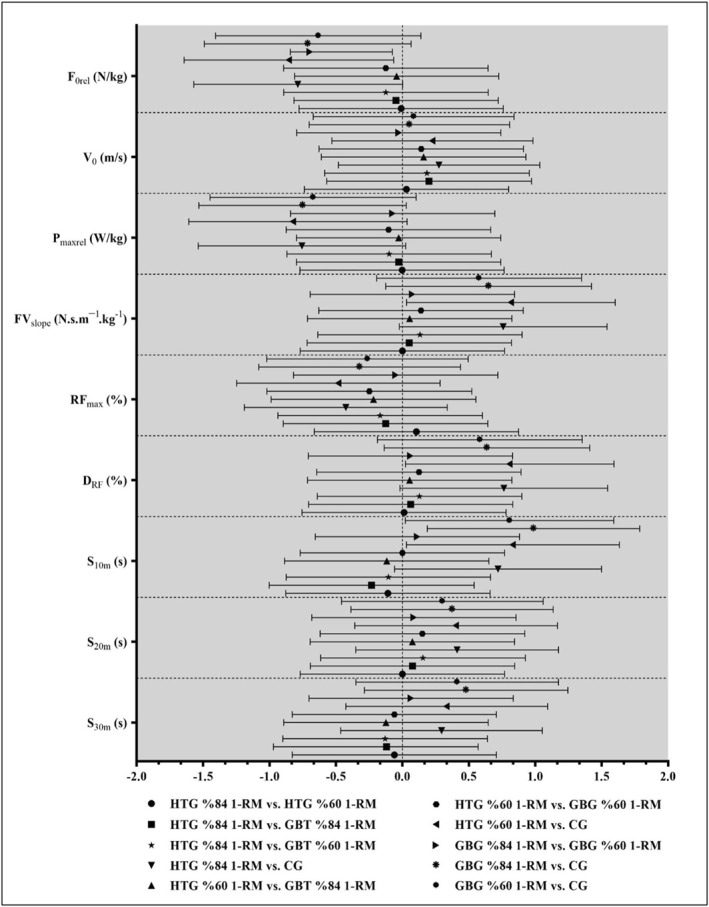
Effect sizes (Hedge's g) for between‐group (post‐PAPE protocols) comparisons (Between‐ES).

According to the results of between‐ES comparisons (Table 3), the GBG 84% 1‐RM protocol had a small effect on V_0_ and S_10m_ compared to the HTG 84% 1‐RM protocol (*g* = 0.201 and *g* = 0.232, respectively). The HTG 60% 1‐RM protocol had a small effect on RF_max_ compared to the GBG 84% and 60% 1‐RM protocols (*g* = −0.217 and *g* = −0.248, respectively). Additionally, the HTG and GBG 84% and 60% 1‐RM protocols had small effects on S_30m_ compared to the CG (*g* = 0.295 to 0.480) (Figure 3).

## 5 Discussion

The results of the study showed that PAPE protocols with HT and GB exercises at 84% and 60% 1‐RM loads had moderate or small effects on HF–V profile components as well as on S_10m_, S_20m_, and S_30m_ performances. Given that sprint is more dependent on horizontal force production rather than vertical force production (Brughelli, Cronin, and Chaouachi 2011), it is expected that HT and GB exercises would significantly impact SP. The obtained data can provide meaningful knowledge for future research and training guidance for coaches and practitioners. First, considering that the potentiating effect depends on the applied load, the results of this study suggest that if coaches and athletes choose to implement this training practice, moderate loads are recommended when applying a CA in adolescent soccer players. These results are consistent with previous studies (I. Dello Iacono et al. 2018; Dello Iacono and Seitz 2018; Rahimi 2007; Wilson et al. 2013). Second, coaches could incorporate the GB exercise into training programs as a CA, providing an alternative tool to help improve both the acceleration phase and kinetics in SP.

The originality of this study lies in the novel inclusion of the GB exercise to induce PAPE effects in adolescent soccer players, setting it apart from previous research that has predominantly focused on HT exercises. This distinction from HT, which primarily targets the vastus lateralis along with the gluteus maximus, adds an innovative dimension to the study by exploring how different muscle activation patterns influence SP. By comparing the effects of GB and HT on SP and HF–V profile components, this study provides new insights into the potential benefits of GB exercises, particularly for players in sports such as soccer where acceleration phases are crucial. This focus on the GB exercise not only broadens the current understanding of muscle‐specific conditioning methods but also suggests alternative strategies for enhancing SP through individualized training programs tailored to specific muscle activation requirements. Consequently, this approach could set a new direction for conditioning research by emphasizing the importance of exercise selection based on the biomechanics of sport‐specific movements.

Previous studies have explained the force production capacity in the HT exercise and compared it with other exercises. The methodology in these studies has primarily relied on electromyographic (EMG) amplitudes recorded. The back squat and HT exercises were compared for their EMG activity in the gluteus maximus, biceps femoris, and vastus lateralis muscles, with HT showing higher muscle activation. Specifically, HT activated the upper gluteus maximus (average: 69.5% vs. 29.4% and peak: 172% vs. 84.9%), lower gluteus maximus (average: 86.8% vs. 45.4% and peak: 216% vs. 130%), and biceps femoris (average: 40.8% vs. 14.9% and peak: 86.9% vs. 37.5%) more than the squat exercise (Contreras et al. 2015). It has also been suggested that the HT is superior to squat and split squat exercises in terms of gluteus maximus activation (ES = 1.08) (Williams et al. 2021). Kennedy et al. (2022) investigated the effects of HT and GB exercises on hip extensor muscles. Their findings indicated that HT elicited greater activation of the vastus lateralis, whereas GB was associated with higher activation levels in the gluteus medius and gluteus maximus muscles (Kennedy et al. 2022). The greatest extensor moment in HT occurs near full‐hip extension (Brazil et al. 2021; Zabaleta Korta and Fernández Peña 2024), whereas a lower hip flexion angle may make the GB exercise more effective for gluteal muscle development. Although the HT and GB exercises target different muscles, they may show similar effects on SP, given that vastus muscles are engaged throughout the sprint and the gluteus medius plays a crucial role in the acceleration phase of sprint (Howard, Conway, and Harrison 2018; Nuell et al. 2021). However, due to the limited research on the GB exercise, this perspective may lack validation and further studies are necessary to confirm its effectiveness.

The findings of this research demonstrate that PAPE protocols with HT and GB exercises have a moderate effect on S_10m_ (*g* = −0.626 to −0.745), a small effect on S_20m_, and a small to trival effect on S_30m_. In the literature, PAPE has a moderate effect (ES = 0.51) on SP (Seitz and Haff 2016). Some studies suggest that PAPE protocols have a significant impact on S_30m_ (de Oliveira et al. 2017; Evetovich, Conley, and McCawley 2015; Petisco et al. 2019), whereas others argue that they do not (Chatzopoulos et al. 2007; Lim and Kong 2013; Mcbride et al. 2005; Vanderka et al. 2016). Additionally, there are studies indicating that PAPE protocols have a significant impact on S_10m_ (Bevan et al. 2010; I. Dello Iacono et al. 2018; Dello Iacono and Seitz 2018). However, a study conducted on rugby players demonstrated that PAPE protocols does not have a significant impact on S_10m_ (Carbone et al. 2020). The ability to produce high muscle force at the start of a sprint is crucial for acceleration and short‐distance SP (de Oliveira et al. 2017). Acceleration performance depends on the magnitude and direction of ground reaction force (GRF) vector associated with sprint kinetics. As velocity increases, an effective application of lower limb force in the horizontal direction is associated with SP (Zisi et al. 2022). The longer ground contact times during maximum sprint, compared to the initial acceleration phase, significantly impact the concentric force production of the knee and hip extensors. This is considered a key factor influencing SP. Current studies indicate that in shorter sprints (10–20 m), which primarily comprise acceleration phases, the correlation between SP and maximal mechanical output development is more evident (Markovic and Mikulic 2010; J. Morin et al. 2012). In this context, it can be hypothesized that the impact of PAPE on muscle force production may influence the acceleration phase of a sprint more than other phases. This emphasizes the importance of focusing on the acceleration phase to enhance SP.

The study observed that PAPE protocols with 84% and 60% 1‐RM loads with HT and GB exercises had a moderate effect on F_0rel_, P_maxrel_, FV_slope_, and D_RF_, whereas having a small effect V_0_ and RF_max_. To our knowledge, no previous research has examined the effects of prior CA protocols on HF–V profile components in adolescent soccer players with at least 2 years of resistance training experience. Galvan et al. (2022) investigated the immediate effects of full squat and HT exercises with 60% and 85% 1‐RM loads on sprint ability in young tennis players. Their findings revealed that HT at 60% 1‐RM influenced S_5m_, *P*_max_, and RF_max_, whereas full squats at 60% 1‐RM affected S_5m_, S_10m_, F_0_, *P*_max_, and RF_max_ (Fernández‐Galván et al. 2022). Participants in Galvan's study were still undergoing maturation, affecting neuromuscular function and physical performance, whereas our study's participants had completed maturation complicating comparisons. In the past decade, several studies have investigated the mechanical determinants of sprint running acceleration through HF–V profile components. The main findings of these studies indicate that SP depends on the average horizontal power and force produced throughout the acceleration phase (Morin et al. 2011, 2012; Rabita et al. 2015). High external loads have a greater impact on F_0_, whereas high training volume primarily influences the V_0_ component. Changes in either F_0_ or V_0_, or both, provide a comprehensive assessment of the neuromuscular state of the muscle directly affecting *P*_max_ as well (Pálinkás et al. 2024). Literature suggests nearly perfect correlations between F_0_ and S_5m_ and S_10m_ and between *P*_max_ and S_10m_ and S_20m_ (Baena‐Raya et al. 2022). As distance and speed increase, *P*_max_ and V_0_ become more critical (P. Samozino et al. 2016). Effective horizontal force (F_*h*_) vector during sprints is essential for effective propulsion and horizontal momentum (Randell et al. 2010; P. Samozino et al. 2016). Athletes who struggle to apply sufficient F_*h*_ at lower velocities—often due to physiological and technical limitations—face disadvantages in on‐field competition. During the initial phases of a sprint, such athletes are unable to produce adequate F_*h*_ leading to a reduced horizontal impulse. Consequently, the maximum speed these athletes achieve is diminished, as it directly correlates with their acceleration capacity (Hicks et al. 2020). Horizontal‐oriented exercises support the application of force in the same direction as seen in the acceleration phase of sprint. During acceleration and maximum speed running, the F_*h*_ vector is expressed in a direction relatively similar to that of the athlete and the direction of force relative to the athlete remains largely the same (Abade et al. 2021; Fitzpatrick, Cimadoro, and Cleather 2019). In this context, considering the specific characteristics of HT and GB exercises, as well as the established effects of PAPE in enhancing maximal voluntary strength, power, or speed post‐conditioning, these findings align well with expectations. Both HT and GB exercises, as horizontally oriented movements, may support the application of the F_*h*_ required during the acceleration phase of sprint. This alignment in movement orientation may have contributed to the observed effects on HF–V profile components, as these exercises are consistent with muscle activation patterns that facilitate F_*h*_ production.

Another finding of our research is that PAPE protocols have a greater impact on D_RF_ (related to the ability to apply force at high speeds) compared to RF_max_ (related to the ability to apply high levels of force at low speeds). D_RF_ is associated with the capacity to apply force at high speeds, whereas RF_max_ indicates the ability to apply high levels of force at lower speeds. In the early acceleration phase, the relationship between RF_max_ and V_0_ defines the direction in which force is applied during the first steps of a sprint, that is, mechanical effectiveness. This mechanical effectiveness is a technical component achieved within approximately 0.3–0.5 s of the sprint (Jiménez‐Reyes et al. 2019). As running speed increases, the slope of the linear decrease in net horizontal force (F_*hnet*_) production per meter (D_RF_) a more detailed understanding of an athlete's capacity to apply F_*h*_ or a potential lack thereof as speed increases (Morin et al. 2015). Mechanical effectiveness is critical in determining how an athlete experiences D_RF_ (Morin and Samozino 2016). Given that D_RF_ is directly related to force application at higher speeds, the conditioning effects of HT and GB exercises may have contributed to improvements in muscle activation and neuromuscular efficiency at these speeds. Therefore, the nature of HT and GB exercises, which closely align with the F_*h*_ demands of sprint, are posited to contribute significantly to improvements in F_*h*_ output as running speed increases.

Force–velocity profiling has gained increasing attention as a tool for creating individualized training prescriptions for athletes (Lindberg et al. 2021). However, in recent years, certain discrepancies have emerged within the literature regarding force–velocity profiling. Central to these discrepancies is the concept of “imbalance.” This concept posits that performance deficiencies are not solely due to inadequate F_0_ or V_0_ capacities; rather, an athlete's strength capacity may be disproportionate to their speed capacity or vice versa (Ettema 2023). Studies have shown that individualized training programs based on vertical force–velocity profiling may be more effective in enhancing jump performance compared to traditional strength/power training methods (Jiménez‐Reyes, Samozino, Brughelli, et al. 2017; Jiménez‐Reyes, Samozino, Pareja‐Blanco et al. 2017). In contrast, Lindberg et al. (2021) reported no significant benefits of individualized training based on vertical force–velocity profiling (Lindberg et al. 2021), and Rakovic et al. (2018) found that individualized sprint training based on the HF–V profiling did not surpass generalized sprint training in improving SP (Rakovic et al. 2018). Although the primary aim of our study was to evaluate the acute effects of PAPE protocols involving different loads and exercises on HF–V profile components—thus lying outside the direct scope of this debate—some recommendations can be derived from our findings. Based on the results of this study, coaches who aim to create individualized training programs based on the HF–V profiling can adopt a complementary approach to include HT and GB exercises in their training programs in line with the targeted capacity, considering that components such as F_0_ and V_0_, which define the athlete's capacity, are physiologically independent from each other (Ettema 2023).

One potential limitation of the current study is the choice of a 0.20 m/s mean propulsive velocity for 1‐RM estimations based on the characteristics of our participant group. de Hoyo et al. (2021) suggested a mean propulsive velocity of 0.25 m/s for estimating 1‐RM in the free‐weight HT exercise among recreational lifters with an 22 ± 2 years (de Hoyo et al. 2021). However, this study also emphasized that mean propulsive velocity values may vary depending on participants' experience and strength levels. Meanwhile, Badillo et al. indicated that for accurate 1‐RM calculations, the mean propulsive velocity should not exceed 0.20 m/s (González‐Badillo, Marques, and Sánchez‐Medina 2011). Based on this information and assuming that both HT and GB exercises are horizontal‐oriented movements with similar movement patterns, a propulsive velocity of 0.20 m/s was chosen for both exercises. This selection was made to align with the age and strength characteristics of our participants, aiming to enhance the relevance and applicability of the findings to our study's specific population. Furthermore, to the best of our knowledge, there is no specific mean propulsive velocity value for the GB exercise in the literature. Additionally, considering the lack of a recorded mean propulsive velocity value for the HT exercise in a group of participants similar to our study, our study highlights the deficiency in this area and may provide a preliminary reference and basis for future research. Another limitation is the absence of EMG activity assessment during HT and GB exercises, which would have facilitated a more comprehensive understanding of muscle engagement during these exercises. Additionally, the lack of sprint kinematics assessment, including variables such as step length, step frequency, step ground contact time, and step flight time, limits the study's ability to provide deeper insights into the determinants of SP. Including these measurements would have contributed to a more thorough evaluation of the specific factors influencing sprint mechanics.

## 6 Conclusion

The results of this study demonstrated that PAPE protocols with 84% and 60% 1‐RM loads with HT and GB exercises can lead to improvements in S_10m_, S_20m_, and S_30m_ as well as some sprint kinetic properties (such as F_0rel_, V_0_, P_maxrel_, FV_slope_, RF_max_, and D_RF_) in the context of their prior CA effects. These results suggest that GB exercises may be an effective alternative to HT exercises for inducing PAPE effects, particularly in enhancing SP and related kinetic properties in soccer players. Given that this study represents the first attempt to implement HT and GB exercises to induce PAPE effects and examine their influence on HF–V profile components, there are gaps in the literature that highlight the need for further more comprehensive research to validate the specific effectiveness of GB exercises. Future research is essential to address these knowledge gaps and to provide a more comprehensive understanding of how PAPE protocols, particularly those incorporating varied exercises, can optimally enhance athletic performance.

## Conflicts of Interest

The authors declare no conflicts of interest.

## Data Availability

The data analyzed during the current study are accessible from the corresponding author on reasonable request.
